# Transfer of the Oral Gonadotropin‐Releasing Hormone Receptor Antagonist Relugolix Into Breast Milk of Healthy Lactating Women

**DOI:** 10.1002/prp2.70067

**Published:** 2025-01-30

**Authors:** Darin B. Brimhall, Yu‐Luan Chen, Sarah Lee, Kazumasa Yoshida, Mike Ufer

**Affiliations:** ^1^ PPD Incorporation—Thermo Fisher Scientific Waltham Massachusetts USA; ^2^ Sumitomo Pharma America Marlborough Massachusetts USA; ^3^ Sumitomo Pharma Co. Ltd. Tokyo Japan; ^4^ Sumitomo Pharma Switzerland GmbH Basel Switzerland

**Keywords:** breast milk, endometriosis, gonadotropin, lactation, pharmacokinetics, relugolix, uterine fibroids

## Abstract

Relugolix is an oral gonadotropin‐releasing hormone receptor antagonist that suppresses sex steroid hormones and is approved as monotherapy for prostate cancer and as a fixed‐dose combination with estradiol/norethindrone for the treatment of endometriosis and uterine fibroids. The aim of this postmarketing study was to determine the pharmacokinetics and quantify the amount of relugolix excreted into breast milk of healthy lactating women. Following a single, oral dose of 40 mg relugolix, breast milk was sampled over 120 h. Pharmacokinetic parameters were determined, including the cumulative amount of relugolix excreted into breast milk to derive the total infant dose. The safety and tolerability of relugolix were also assessed. Eight healthy lactating women were enrolled and completed the study per protocol. Relugolix was safe and well tolerated based on adverse events and other safety data. It was excreted into breast milk with a median time to peak concentration (*t*_max_) of 5.81 h and a geometric mean peak concentration (*C*_max_) of 15.7 ng/mL, similar to corresponding plasma data from previous clinical studies. The mean cumulative amount of relugolix excreted was 0.0051 mg over 24 h and 0.0067 mg over 120 h, corresponding to 0.0128% and 0.0167% of the maternal dose, respectively. The body weight‐adjusted relative daily infant dose of approximately 0.25% suggests a 400‐fold lower newborn than maternal relugolix exposure. Relevant effects of relugolix on the breastfed child appear unlikely given its limited excretion into breast milk of lactating women but cannot be fully excluded in the absence of infant safety data.

## Graphical abstract

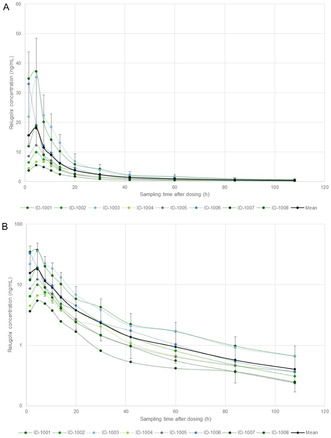

## 1 Introduction

Relugolix is an oral gonadotropin‐releasing hormone receptor antagonist that competitively binds to receptors in the anterior pituitary and leads to a reversible, dose‐dependent suppression of luteinizing hormone and follicle‐stimulating hormone, thereby lowering levels of sex steroid hormones such as testosterone, estradiol, and progesterone [1, 2].

This mechanism counteracts the proliferative effects of (i) testosterone on prostate cancer cells in men and (ii) estradiol on endometrial tissue and uterine fibroids in women, leading to the improvement of moderate to severe pain in patients with endometriosis and to a significant reduction of heavy menstrual bleeding in patients with uterine fibroids, respectively [3, 4]. Therefore, relugolix is approved for the treatment of both endometriosis and uterine fibroids under the trade names MYFEMBREE in the United States and RYEQUO in Europe. It is available as a fixed‐dose combination (FDC) product for once‐daily oral use and contains 40 mg relugolix, 1.0 mg estradiol (E2), and 0.5 mg norethindrone acetate (NETA). The combination with E2 and NETA is intended to maintain estradiol levels within the physiologic range, thereby minimizing hypoestrogenic side effects and preserving bone mineral density [1].

The pharmacokinetic (PK) characteristics of relugolix in plasma have been thoroughly studied in a comprehensive clinical pharmacology study program. In short, relugolix is rapidly absorbed with a time to peak plasma concentration (*t*_max_) of approximately 2 h when administered as part of the FDC product. It is a substrate of P‐glycoprotein (P‐gp) and has a low oral bioavailability of 12%. The effective elimination half‐life of relugolix is approximately 24 h, consistent with a two‐fold accumulation ratio upon multiple‐dose administration. Its elimination mainly occurs in the form of metabolites that are formed by CYP2C19 and CYP3A4, and via other minor metabolic pathways [1, 5, 6].

In lactating rats, relugolix was present in breast milk following the administration of a single oral dose, and its concentrations exceeded those in plasma by up to 10‐fold. In the absence of human data on the potential excretion of relugolix into breast milk, breastfeeding is not recommended during relugolix combination therapy according to current labeling information in the United States [7] and is contraindicated in Europe [8]. However, there may be less of a clinical need for treatment with relugolix in lactating patients with endometriosis or uterine fibroids due to the physiological prolactin‐mediated suppression of estradiol levels during lactation [9]. Nevertheless, this clinical lactation study was requested as a postmarketing requirement by the US Food and Drug Administration (FDA) in view of available animal data and in consideration of the target patient population comprising young women of childbearing potential to provide product labeling information on potential infant exposure.

Here, we report on the results from this postmarketing study that assessed the potential transfer of relugolix from blood into breast milk of healthy lactating women following oral administration of a single therapeutic dose of 40 mg relugolix.

## 2 Material and Methods

The study was conducted at PPD Development in Las Vegas, NV, USA, in compliance with regulatory requirements and the principles outlined in the Declaration of Helsinki and International Council for Harmonization (ICH) guideline for Good Clinical Practice. Approval of the study protocol was obtained from the institutional review board (Advarra, 6100 Merriweather Drive, Suite 600, Columbia, MD 21044, USA) and the US FDA. All participants signed an informed consent form prior to any study assessment.

### 2.1 Study Design

**FIGURE 1:**
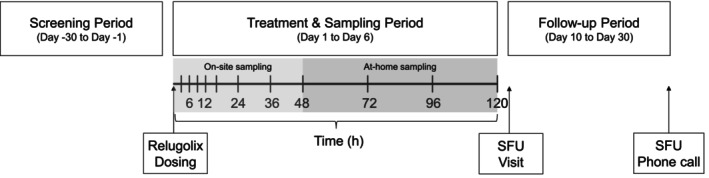
Study design. SFU, safety follow‐up.

This was a prospective, single‐center, open‐label, single‐dose, Phase 1 study to determine the PK of relugolix in breast milk of eight healthy, lactating women (Figure 1). In addition, the safety and tolerability of relugolix were assessed.

Study participants were screened for study eligibility at a screening visit within 30 days prior to dosing and admitted to the clinical research unit for predose baseline assessments on the day prior to dosing. On Study Day 1, a single, oral dose of 40 mg relugolix was administered to each study participant, followed by serial breast milk collection and safety assessments. Study participants were confined to the unit for at least 48 h after dosing. Thereafter, they were discharged and continued breast milk collection at home. Each study participant returned to the unit for a safety follow‐up visit approximately 10 days after dosing and received a safety follow‐up phone call approximately 30 days after dosing.

### 2.2 Study Population

Healthy, non‐smoking, lactating women aged 18–45 years with a body mass index between 18.5 and 34.0 kg/m^2^ were eligible. Each study participant was ≥ 4 weeks postpartum on the day of dosing and determined to be healthy based on medical history, physical examination as well as clinical laboratory, vital sign, and electrocardiogram data. Concomitant drug use was prohibited from ≥ 28 days (P‐gp inhibitors/inducers) or from ≥ 14 days (all other drugs) prior to dosing until ≥ 10 days after dosing unless required for treatment of adverse events.

### 2.3 Breast Milk Sampling

Each study participant temporarily discontinued breastfeeding for ≥ 10 days after dosing. Breast milk samples were collected predose and up to 120 h postdose during prespecified intervals (i.e., 0–3, 3–6, 6–9, 9–12, 12–16, 16–24, 24–36, 36–48, 48–72, 72–96, and 96–120 h) using a double electric breast pump (Medela Sonata, Illinois, USA).

At each collection interval, both breasts were emptied completely. All breast milk produced was collected (inclusive of foremilk and hindmilk) and milk samples of each collection interval were pooled. The total volume of breast milk collected at the specified intervals was determined, and the start and finish times of each collection were recorded. Aliquots from each interval were drawn, stored at −80°C or lower, and used for analysis.

### 2.4 Bioanalytical Sample Assays and PK Analysis

Relugolix concentrations in breast milk were determined using a validated liquid chromatography–tandem mass spectrometry (LC–MS/MS) method based on a sample volume of 0.100 mL. The bioanalytical method used a *d*_*6*_‐relugolix as the internal standard (IS) and ethyl acetate (0.6 mL) for sample extraction at pH 10 ammonium acetate buffer, coupled with an Acquity UPLC BEH C8 column (50 × 2.1 mm, 1.7 μm) for separation followed by MS/MS detection of (*m*/*z*) 624.2➔ 548.2 for relugolix and 630.2 ➔ 548.2 for IS in the positive ion mode. The collision gas was nitrogen, and the collision energy was 35 eV. The source temperature was 650°C. In this method, a gradient mobile phase was utilized as follows: 0–0.20 min with 36% B, 0.20–1.40 min from 36% to 46% B, 1.40–1.50 min from 46% to 100% B, holding up to 2.20 min with 100% B, then back down to 36% B at 2.22 min, and reequilibrating through 2.80 min (where mobile phase A was 0.1% formic acid in H_2_O and mobile phase B was 0.1% formic acid in methanol). The retention times for both relugolix and IS were ~ 1.1 min. The validated calibration range was 0.05–50.0 ng/mL with a lower limit of quantification (LLOQ) of 0.05 ng/mL. The breast milk method was validated according to the ICH M10 guideline for bioanalytical method validation and study sample analysis [10]. The specifications were the same as those in the previously validated plasma method in terms of calibration curve range, sensitivity, sample volume, sample extraction, storage conditions, and LC–MS/MS monitoring parameters [5].

Precision and accuracy were evaluated by quantification of quality control pools at four concentrations spanning the calibration range. Both were acceptable as determined by a percent coefficient of variation (CV%) below 15% and a mean percent difference from the theoretical concentration within ±15%. All breast milk samples had relugolix concentrations within the validated method range. Twenty‐two (22) samples were selected for incurred sample reanalysis evaluation, and 100% met the acceptance criteria. All samples were analyzed within the established sample frozen storage stability. The amount of relugolix excreted during each collection interval was calculated by multiplying the measured concentration by the total volume collected.

The PK parameters of relugolix in breast milk were determined by noncompartmental analysis based on the midpoint approach (i.e., midpoint between actual start and end time of each prespecified collection interval) using Phoenix version 8.3 (CERTARA, Pharsight Inc., Princeton, NJ, USA).

### 2.5 Sample Size Estimation and Statistical Data Analysis

Based on the inter‐ and intrasubject PK variability of relugolix in previous Phase 1 studies [5, 6] and in accordance with the applicable FDA guidance on clinical lactation studies, a minimum sample size of eight lactating women was considered sufficient. Descriptive statistics was applied for demographic, safety and tolerability data.

### 2.6 Nomenclature of Targets and Ligands

Key protein targets and ligands in this article are hyperlinked to corresponding entries in http://www.guidetopharmacology.org, the common portal for data from the IUPHAR/BPS Guide to PHARMACOLOGY [11] and are permanently archived in the Concise Guide to PHARMACOLOGY 2019/20 [12].

## 3 Results

### 3.1 Demographics, Baseline Characteristics, and Disposition Data

Eight healthy lactating women were enrolled and completed the study per protocol. They had a mean age of 31.0 years (range: 26–37 years), mean height of 1.64 m (1.57–1.70 m), mean body weight of 68.1 kg (52.3–79.5 kg), and mean BMI of 25.2 kg/m^2^ (19.4–29.9 kg/m^2^). Four participants were White, two were Asian, one was Black or African American, and one was multiracial.

### 3.2 PK Data

**FIGURE 2:**
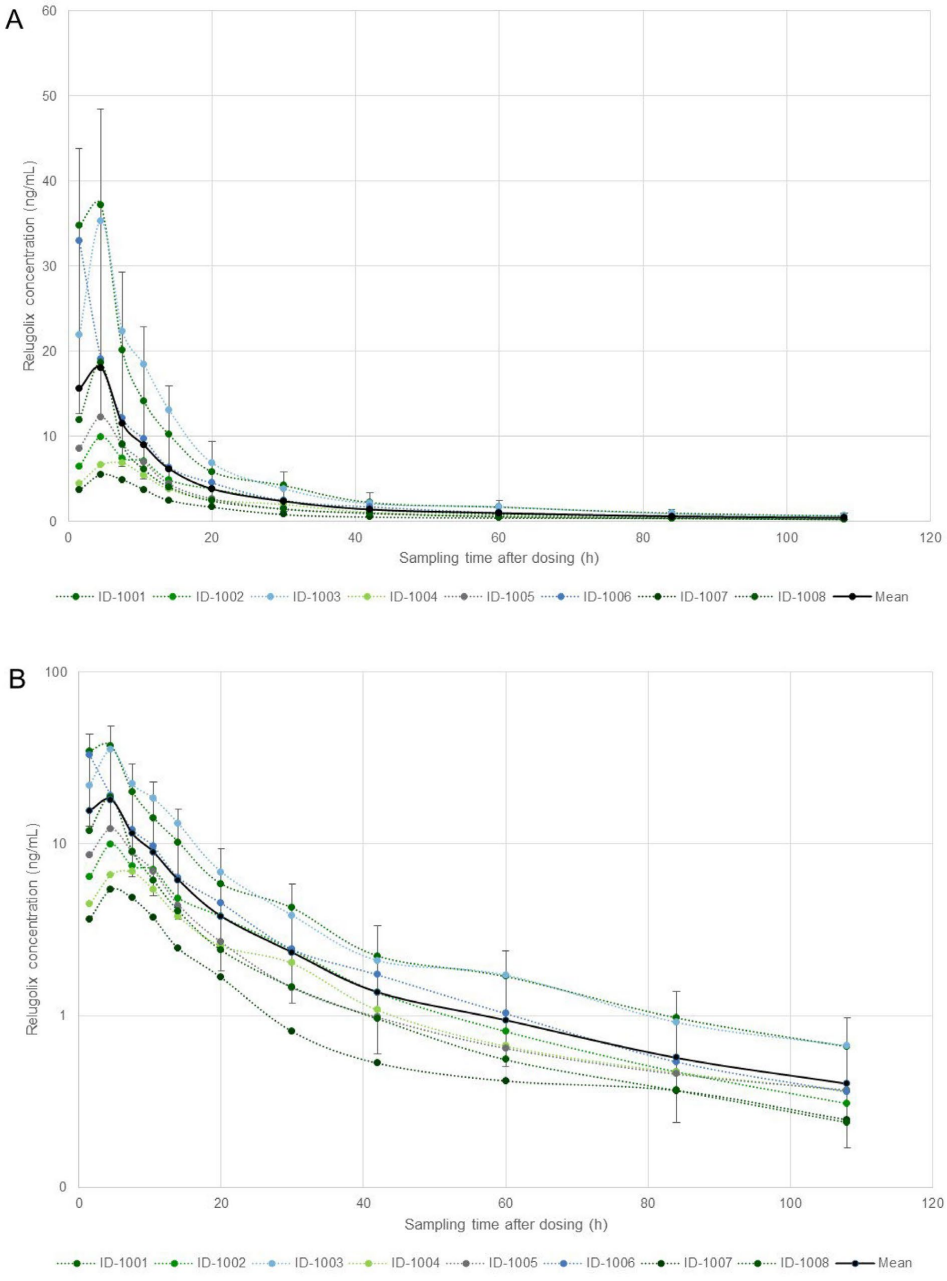
Relugolix concentration/time profiles in breast milk of lactating women. Mean (SD) (solid line) and individual (dotted lines) concentration/time profiles are displayed for each study participant (ID‐1001‐ID‐1008) as linear (A) or semi‐logarithmic plot (B). Samples were collected over the following collection intervals: predose, 0–3, 3–6, 6–9, 9–12, 12–16, 16–24, 24–36, 36–48, 48–72, 72–96, and 96–120 h postdose. Concentrations are plotted at the midpoint of each interval.

Breast milk sampling was completed until 120 h after dosing by each of the eight enrolled participants. Relugolix was quantifiable up to the last collection interval in each participant following a single, oral dose of 40 mg. The mean milk concentration–time profile of relugolix is presented in Figure 2. The PK parameters and infant exposure parameters are summarized in Tables 1 and 2, respectively.

**TABLE 1:** Pharmacokinetic data of relugolix in healthy premenopausal women following a single, oral dose of 40 mg.

PK parameter (unit)	
*C*_max_ (ng/mL)	15.7 (88.6)
AUC_0–*t*_ (ng·h/mL)	277 (59.7)
AUC_0–∞_ (ng·h/mL)	297 (56.3)
*t*_max_ (h)	5.81 (2.79–8.83)
*t*_1/2_ (h)	33.1 (24.4)

*Note:* In plasma, median *t*_max_ ranged from 1.50 to 2.26 h and geometric mean values of *C*_max_ ranged from 9.36 to 16.1 ng/mL, AUC_0–*t*_ from 84.4 to 152 ng·h/mL, AUC_0–∞_ from 93.6 to 161 ng·h/mL, and *t*_1/2_ from 15.9 to 56.6 h based on data from two previously completed Phase 1 studies in healthy premenopausal women following fasted, oral administration of 40 mg relugolix (TAK‐385‐101, MVT‐601‐041) [5].

Abbreviations: AUC_0–*t*_, area under the concentration/time curve up to the last sampling time point; AUC_0–∞_, area under the concentration/time curve extrapolated to infinity; *C*_max_, maximum concentration; CV, coefficient of variation; FE, food effect; n.a., not applicable; SAD, single ascending dose; *t*_1/2_, elimination half‐life; *t*_max_, time to maximum concentration.

**TABLE 2:** Derived infant exposure parameters of relugolix following a single, oral, maternal dose of 40 mg.

Exposure parameter (unit)	Mean (SD)	Median (range)
Daily infant dose (mg)	0.0051 (0.0048)	0.0032 (0.0004–0.0134)
Total infant dose (mg)	0.0067 (0.0059)	0.0044 (0.0006–0.0163)
Fraction excreted over 24 h (%)	0.0128 (0.012)	0.0080 (0.0010–0.0336)
Fraction excreted over 120 h (%)	0.0167 (0.0148)	0.0110 (0.0016–0.0408)
Relative daily infant dose (%)^a^	0.2499 (0.2338)	0.1559 (0.0187–0.6534)
Relative total infant dose (%)	0.3244 (0.2889)	0.2133 (0.0311–0.7942)

*Note:* Exposure parameters are provided as arithmetic mean (SD) and median (range). Daily infant dose = amount excreted over 24 h; total infant dose = amount excreted over 120 h; fraction excreted over 24 h = daily infant dose/maternal dose; fraction excreted over 120 h = total infant dose/maternal dose; relative daily infant dose = fraction excreted over 24 h × (maternal/infant body weight) × 100; and relative total infant dose = fraction excreted over 120 h × (maternal/infant body weight) × 100. Relative daily and total infant doses were calculated based on the maternal dose of 40 mg and an assumed infant body weight of 3.5 kg corresponding to a newborn and a mean maternal body weight of 68.1 kg, as determined in this study.

^a^ The relative daily infant dose may also be calculated according to the following alternative equation [13]: Relative daily infant dose (%) = ([AUC_0‐24_/24 h] × 150 mL/kg/day/[maternal dose/maternal weight]) × 100 = 0.2947%. Considering the two‐fold accumulation ratio of relugolix, the derived relative daily infant dose would increase by two‐fold in the context of multiple dosing.

A geometric mean *C*_max_ of 15.7 ng/mL was reached at a median *t*_max_ of 5.81 h. Thereafter, relugolix concentrations decreased with a mean *t*_1/2_ of 33.1 h. A geometric mean AUC_0–*t*_ of 277 ng·h/mL and AUC_0–inf_ of 297 ng·h/mL was determined.

The median cumulative amount of relugolix excreted in breast milk over 24 h (i.e., daily infant dose) and 120 h (i.e., total infant dose) after dosing was 0.0051 and 0.0067 mg, respectively. Accordingly, the fraction of relugolix excreted over 24 h (i.e., daily infant dose) and 120 h (i.e., total infant dose) after dosing was 0.0128% and 0.0167%, respectively (Table 2).

The mean relative daily and total infant doses of 0.2499% and 0.3244%, respectively, were calculated based on an assumed infant body weight of 3.5 kg, corresponding to a newborn child, and based on the mean maternal body weight of 68.1 kg determined in this study (Table 2).

The mean total daily breast milk volumes were generally similar between the in‐house collection period (i.e., Day 1: 522.1 mL [SD 347.4]; Day 2: 466.9 mL [SD 317.9]) and the outpatient collection period (i.e., Day 3: 392.3 mL [SD 322.2]; Day 4: 377.3 mL [SD 360.2]; Day 5: 399.9 mL [SD 309.9]).

### 3.3 Safety and Tolerability Data

A single oral dose of 40 mg relugolix was safe and well tolerated in this study population of healthy lactating women. No serious or severe adverse events were reported.

Overall, four adverse events were reported by three participants. Two of these adverse events were treatment emergent, that is, ligament sprain (not related; Grade 2) and abdominal discomfort (related; Grade 1). The latter event occurred on the day of dosing and resolved in less than 6 h without pharmacological treatment. The other two adverse events were not treatment emergent, as they occurred prior to dosing, that is, headache reported by two participants.

There were no clinically relevant changes in other safety variables, that is, clinical laboratory, vital sign, or electrocardiography data.

## 4 Discussion

In this single‐center, Phase 1 study, the PK of the gonadotropin‐releasing hormone receptor antagonist relugolix was determined in breast milk of healthy lactating women following single, oral dose administration. It provides information about the amount of relugolix transferred into breast milk and the potential relugolix exposure of the breastfed infant.

Drug labeling information often lacks clinical data about the extent of drug excretion into breast milk, and as such, it is commonly recommended (i) to abstain from using medications while breastfeeding due to uncertainty about possible adverse effects on their infants or (ii) to discontinue breastfeeding if drug treatment is required [14]. Health authorities have recognized that data from clinical lactation studies may be useful to health care providers and breastfeeding women to allow for informed risk/benefit decisions [15]. Accordingly, lactation studies are occasionally requested as a postmarketing requirement by the US FDA to derive appropriate product labeling information [16, 17].

The study design was compliant with the applicable FDA draft guidance on clinical lactation studies (Figure 1) [18]. Although the FDC product (40 mg relugolix, 1 mg E2, and 0.5 mg NETA) is approved for the treatment of endometriosis and uterine fibroids, relugolix alone was administered in the present study to prevent any estrogen‐mediated reduction of breast milk production [19]. The selected oral dose of 40 mg relugolix corresponds to the therapeutic once‐daily dose approved for the treatment of endometriosis and uterine fibroids [20, 21]. Although relugolix shows a two‐fold accumulation upon multiple dosing consistent with its effective elimination half‐life of approximately 24 h, a single‐dose study was considered adequate because the PK of relugolix is not time dependent and multiple‐dose exposure can be well predicted based on single‐dose data utilizing population PK modeling [22].

Following oral administration, relugolix quickly transitioned into breast milk of each study participant (Table 1). *C*_max_, *t*_max_, and *t*_1/2_ were comparable to plasma data from two previously completed Phase 1 studies in healthy premenopausal women following fasted, oral administration of 40 mg relugolix [5]. However, relugolix exposure (i.e., AUC_0–*t*_ and AUC_0–inf_) was approximately two‐ to three‐fold higher in breast milk than in plasma [5]. This may be due to the chemical properties of relugolix, which is an organic amine with a pKa of 8.63 [23]. As the pH of breast milk is lower than that of plasma (i.e., approximately 7.2 vs. 7.4), the solubility of relugolix increases after transition into breast milk, and it may hence be subject to ion trapping, which is a well‐known phenomenon for drugs with a pKa value greater than 7.2 [24].

In a previous rat study, relugolix concentrations were up to 10‐fold higher in breast milk than in plasma following administration of a single oral dose [7]. This exemplifies that the milk/plasma ratio determined in animal models may not always well predict data in humans [15]. A higher milk/plasma ratio in rodents than in humans is often observed, but the underlying mechanistic reasons for this phenomenon remain unclear. It is known that the lipid content of breast milk is approximately two‐fold higher in rats than in humans [25]. As such, relugolix may transition to a greater extent into rat than human breast milk, given its lipophilicity [26]. Physiologically based PK (PBPK) modeling has more recently been suggested as a tool to improve the prediction of drug concentrations in breast milk during breastfeeding based on the physicochemical properties of the investigational drug, mechanisms of drug transport, infant physiology and breast milk composition [27, 28, 29].

The current labeling information includes information about the presence of relugolix in breast milk of lactating rats and highlights the absence of corresponding human data [7, 8]. In terms of drug product labeling, the data from this clinical lactation study should now prevail over previously determined rodent data, as the FDA's Pregnancy and Lactation Labelling Guidance states that animal data must not be included in the product label when human data are available [30].

The goal of any lactation study would be to inform on the risk to the infant arising from breastfeeding due to unintended exposure to any drug used by the lactating mother. However, a limitation of most lactation studies, including the present one, is the restriction to PK data obtained from the mother, whereas mother–infant pair studies that also include safety data collection in exposed infants are rarely conducted. Therefore, an appropriate risk assessment requires considerations about the mechanism of action, pharmacodynamic effects and safety profile of the drug concerned in addition to the exposure data. Nevertheless, milk‐only PK studies currently represent the default approach recommended by the FDA unless the PK characteristics of the drug under investigation are unknown in women or there is any concern for drug accumulation in breast milk [18].

Despite these limitations, the calculated infant exposure is an important outcome measure of any clinical lactation study. In the present study, the mean cumulative amount of relugolix excreted into breast milk over 120 h was 0.0067 mg (i.e., total infant dose), which corresponds to 0.0167% of the maternal dose. Approximately 75% of this total amount was recovered during the first 24 h after dosing, corresponding to a daily infant dose of 0.0051 mg. The body weight–adjusted relative daily infant dose was approximately 0.25%, assuming an infant body weight of 3.5 kg, corresponding to a newborn. This is well below the often‐cited reference threshold of 5%–10% above which the infant exposure may pose a safety risk [13, 31]. Considering the two‐fold accumulation ratio of relugolix, the relative daily infant dose would still remain well below this threshold in the context of multiple dosing.

In conclusion, the oral gonadotropin‐releasing hormone receptor antagonist relugolix transitions into human breast milk. However, the amount of relugolix excreted into breast milk was low, indicated by a body weight‐adjusted relative daily infant dose of approximately 0.25%, suggesting a 400‐fold lower infant than maternal exposure. Therefore, relevant effects of relugolix on the breastfed child appear unlikely but cannot be fully excluded in the absence of infant safety data.

## Author Contributions

All authors reviewed the manuscript and approved its content. D.B.B. contributed to clinical study conduct as the principal investigator. Y.‐L.C. and S.L. were responsible for all bioanalytical aspects of the study. K.Y. analyzed the data. M.U. served as the sponsor's clinical study lead and wrote the manuscript.

## Ethics Statement

Approval of the study protocol was obtained from the institutional review board (Advarra, IRB#00000971, 6100 Merriweather Drive, Suite 600, Columbia, MD 21044, USA) under the approval number Pro00074859.

## Consent

All participants signed an informed consent form prior to any study assessment.

## Conflicts of Interest

Y.‐L.C., K.Y., and M.U. are employees of Sumitomo Pharma. S.L. is a former employee of Sumitomo Pharma. D.B.B. served as the principal investigator.

## Data Availability

The data of this study are available on reasonable request from the corresponding author. The data are not publicly available due to privacy and ethical restrictions.
